# Construction of a prediction model for axillary lymph node metastasis in stage cN0 hormone receptor-positive breast cancer: based on interpretable machine learning methods

**DOI:** 10.3389/fonc.2026.1763228

**Published:** 2026-02-03

**Authors:** Wenyan Liu, Zhijun Ma, Yufei Wang, Qishuai Chen, Liu Wang, Jiuqing Chi

**Affiliations:** 1 Clinical Medicine College, Graduate School of Qinghai University, Xining, Qinghai, China; 2 Department of Oncology Surgery, Qinghai University Affiliated Hospital, Xining, Qinghai, China

**Keywords:** axillary lymph node metastasis, breast cancer, hormone receptor positivity, machine learning, predictive modeling

## Abstract

**Background:**

Accurately predicting axillary lymph node metastasis (ALNM) preoperatively is crucial for optimizing management in patients with clinically node-negative (cN0) hormone receptor-positive (HR+) breast cancer (BC).

**Methods:**

We retrospectively analyzed 816 cN0 HR+ BC patients (2016-2024). Data from 2016-2023 (n=726) were randomly assigned to a training set (n=503) or an internal test set (n=223) in a 7:3 ratio. Patients treated in the most recent year, 2024 (n=90), were reserved as a held-out temporal validation set. Following feature selection via Recursive Feature Elimination (RFE), five machine learning models—XGBoost, Random Forest, Logistic Regression, Support Vector Machine, and K-Nearest Neighbors (KNN)—were developed. Performance was assessed by the area under the receiver operating characteristic curve (AUC) and decision curve analysis (DCA). The optimal model was interpreted using SHapley Additive exPlanations (SHAP).

**Results:**

The incidence of ALNM was 30.9%. The KNN model demonstrated optimal performance, achieving an AUC of 0.898 (95% CI: 0.857–0.939) in the test set and 0.774 (95% CI: 0.655–0.892) in the external validation set. DCA indicated that the KNN model provided the highest net clinical benefit within the 30%–65% threshold probability range. SHAP analysis identified parity as the most critical predictor, followed by age, tumor location, menopausal status, tumor diameter, lymphocyte count, platelet count, alpha-fetoprotein (AFP), neutrophil count, and carcinoembryonic antigen (CEA).

**Conclusion:**

The KNN model, integrated with the SHAP interpretability framework, shows favorable performance, interpretability, and clinical applicability for predicting ALNM in cN0 HR+ BC, offering a valuable tool for preoperative risk assessment and individualized decision-making.

## 1 Introduction

Breast cancer (BC) is the most commonly diagnosed malignancy and a leading cause of cancer-related mortality among women worldwide (1). Clinical management is guided by molecular subtypes, with hormone receptor-positive (HR+) BC (encompassing Luminal A and B) being the most prevalent, accounting for approximately 70% of cases (2). Luminal A tumors tend to follow a more indolent course, whereas Luminal B tumors are associated with a higher risk of recurrence (3). This intrinsic diversity results in a broad spectrum of metastatic potential, rendering the preoperative distinction between patients at genuine low risk (who may be candidates for de-escalated axillary surgery) and those with occult high-risk disease particularly challenging yet critical within the HR+ population. Consequently, management strategies for HR+ patients require greater refinement, with accurate assessment of axillary lymph node status being paramount for prognosis and therapeutic decision-making.

Axillary lymph node metastasis is one of the most important independent prognostic factors in breast cancer, directly influencing disease staging, treatment selection, and subsequent adjuvant therapy decisions (4). For patients with negative clinical lymph nodes (cN0), that is, those whose regional lymph node metastasis is not found on preoperative palpation and imaging (5), sentinel lymph node biopsy (SLNB) is a conventional pathological staging method. However, a significant proportion (approximately 20–40%) of cN0 patients are found to have nodal metastases on SLNB, many of whom may not benefit additionally from completion axillary lymph node dissection (ALND), potentially facing overtreatment (6, 7). The development of reliable tools to preoperatively stratify ALNM risk in cN0 patients, particularly within the large HR+ subgroup, is therefore clinically relevant.

Currently, preoperative prediction of ALNM primarily relies on imaging evaluations (such as ultrasound and MRI) and prediction nomograms based on clinical and pathological characteristics (8, 9). These methods have limitations, including variable accuracy and a lack of specificity for the HR+ subtype. Machine learning (ML) offers a potential avenue for improving prediction by modeling complex relationships in multi-dimensional data (10). While several studies have applied ML to ALNM prediction in BC (11, 12), many include all subtypes or lack validation in separate cohorts. Furthermore, the interpretability of ML models remains a barrier to clinical translation (13).

To address these considerations, this study aimed to develop a HR+ cN0-stage specific prediction model for ALNM using interpretable ML methods. Our objectives were: (1) to construct and compare multiple ML algorithms based on preoperative clinicopathological data; (2) to evaluate the temporal generalizability of the optimal model using a cohort from a subsequent time period; and (3) to employ the SHAP (SHapley Additive exPlanations) framework to interpret the model’s predictions, thereby providing actionable insights for clinicians. This approach may significantly enhance preoperative risk assessment and individualized treatment planning for this patient population.

## 2 Materials and methods

### 2.1 Data split and validation strategy

Patients diagnosed between January 2016 and December 2023 were included in the model development cohort. This cohort was randomly divided into a training set (70%, n=503) and a test set (30%, n=223) for model development, hyperparameter tuning, and initial evaluation.

To assess the model’s performance over time and reduce overfitting, an independent temporal validation cohort was established. This cohort comprised all consecutive patients meeting the same inclusion criteria from the subsequent and non-overlapping period of January 2024 to December 2024 (n=90). Among these 90 patients, 28 cases (31.1%) had ALNM, a finding consistent with the ALNM incidence rate (30.9%) observed in the development cohort. Crucially, this temporal validation set was locked before model development and was used strictly for the final, one-time evaluation of the selected model. No data from this 2024 cohort were used in any phase of model training or parameter tuning.

This study complied with the ethical principles of the Declaration of Helsinki for medical research involving human subjects and was approved by the Ethics Committee of Qinghai University Affiliated Hospital (approval No. P-SL-2024-406). Due to the retrospective design of this study, the Ethics Committee waived the requirement for informed consent. This study adhered to the Transparent Reporting of a Multivariable Prediction Model for Individual Prognosis or Diagnosis (TRIPOD) guidelines to ensure transparency and scientific rigor in study design, implementation, and reporting.

Inclusion Criteria: (1) Female patients aged ≥18 years. (2) Preoperative core needle biopsy pathology confirming: (a) invasive breast carcinoma, and (b) HR+ status (defined as estrogen receptor [ER]-positive and/or progesterone receptor [PR]-positive). (3) Preoperatively assessed as clinically node-negative (cN0 stage) by physical examination and imaging. (4) Underwent primary surgical treatment (mastectomy or breast-conserving surgery) with pathological evaluation of axillary nodal status via SLNB and/or ALND. (5) Availability of complete preoperative data required for modeling.
Exclusion Criteria: (1) Male breast cancer. (2) Inflammatory breast cancer, clinical T4-stage tumors (with chest wall/skin invasion), or evidence of distant metastasis (stage IV disease) at initial diagnosis. (3) Bilateral synchronous or multicentric BC. (4) History of any other invasive malignancy within the past 5 years prior to the current BC diagnosis. (5) Receipt of any form of neoadjuvant therapy (chemotherapy, endocrine therapy, targeted therapy, or radiotherapy) prior to surgery. (6) Preoperative biopsy pathology suggesting non-invasive carcinoma (e.g., ductal carcinoma *in situ*) or microinvasive carcinoma only. (7) Missing or incomplete key preoperative data necessary for model construction.

### 2.2 Predictors and outcome variable

1. Clinical Characteristics: Age at diagnosis, BMI, menopausal status, marital status, parity.
2. Preoperative Core Needle Biopsy Pathology: Estrogen Receptor (ER) Status, Progesterone Receptor (PR) Status, Human Epidermal Growth Factor Receptor 2 (HER2) Status, Ki-67 Index, Histological Grade.
3. Hematologic Data: Alpha-Fetoprotein (AFP), Carcinoembryonic Antigen (CEA), Neutrophil Count, Lymphocyte Count, Platelet Count.
4. Preoperative Imaging Characteristics: Tumor location and maximum diameter of the primary tumor as determined by preoperative breast ultrasound.

Primary Outcome: ALNM status, determined by postoperative histopathological examination of excised sentinel lymph nodes or axillary lymph nodes, which serves as the gold standard.

### 2.3 Pathology interpretation criteria

1. Histological grading: Assessed based on glandular formation proportion, nuclear pleomorphism, and mitotic count. The sum of these three scores categorizes tumors as follows: 3–5 points = Grade I (well-differentiated); 6–7 points = Grade II (moderately differentiated); 8–9 points = Grade III (poorly differentiated) (14).
2. ER and PR interpretation criteria: Nuclear staining in <1% of tumor cells is negative; ≥1% nuclear staining is positive (1–10% = low-positive expression). BC is classified as HR+ if tumor cells show ER and/or PR positivity (15).
3. HER-2 interpretation criteria: For HER-2 immunohistochemical (IHC) staining, IHC 3+ denotes positivity; 0 or 1+ denotes negativity; IHC 2+ necessitates *in situ* hybridization (ISH) confirmation. Fluorescence *in situ* hybridization (FISH)-positive results indicate gene amplification, confirming HER-2 positivity; FISH-negative results are interpreted as HER-2 negative.
4. Ki-67 interpretation criteria: Nuclear brown staining (of any intensity) is defined as Ki-67 positivity. The percentage of Ki-67-positive nuclei among tumor cells is recorded quantitatively (16).
5. P53 interpretation criteria: Nuclear brownish-yellow granular staining is defined as P53 positivity. Semi-quantitative assessment: Ten random high-power fields (×400) are selected to quantify positive cell proportion: <10% = negative (-); 11–30% = weak positive (+); 31–50% = moderate positive (++); >50% = strong positive (+++). For this study, P53 positivity is defined as ≥11% positive cells.
6. Axillary lymph node positivity: Axillary lymph nodes are considered positive if histopathological examination identifies macrometastases, micrometastases, or isolated tumor cells (ITCs).
7. cN0 definition: Preoperative clinical lymph node status was assessed in all patients. cN0 is defined as the absence of regional lymph node metastases detected by imaging or clinical examination (5).

### 2.4 Data preprocessing

The missing values in the dataset were handled using Multiple Imputation by Chained Equations (MICE, implemented via the mice package in R). MICE is an iterative algorithm that fills missing values for each variable by establishing a series of chained regression models. The imputation model incorporated all variables required for subsequent modeling (including clinical features, pathological indicators, laboratory tests, imaging characteristics, and outcome variables). Notably, the outcome variable had no missing values prior to imputation. The *mice()* function was used with default settings to generate 5 imputed datasets, and the third imputed dataset (complete(imp, 3)) was selected for subsequent analysis. Use the md.pattern(mydata) function to visualize missing data and confirm that missing values are randomly distributed among variables, with no concentration in specific subgroups. In addition, continuous variables were standardized, and categorical variables were factorized. Crucially, to prevent data leakage, all preprocessing steps strictly adhered to a sequential timeline and workflow:

1. Initial Split: The primary cohort (patients from 2016–2023, n=726) was first randomly split into a training set (70%, n=503) and a test set (30%, n=223).
2. Class Imbalance Handling: To address class imbalance, the Random Over-Sampling Examples (ROSE) technique was applied exclusively to the training set. The test set was kept completely untouched and remained in its original imbalanced state to ensure an unbiased evaluation (17).
3. Independent Validation Set: The temporal validation cohort (patients from 2024, n=90) was completely isolated and locked prior to analysis. It underwent no resampling or preprocessing based on the development cohort and was used solely for final model evaluation.

### 2.5 Feature selection

Recursive Feature Elimination (RFE) was utilized to identify the optimal subset of predictive features. RFE iteratively removes features with the lowest contribution to model performance, systematically quantifying each feature’s importance while mitigating the risk of overfitting. To prevent information leakage, feature selection was restricted to the training set exclusively, with five-fold cross-validation employed to ensure model robustness. Ultimately, RFE identified 18 potential predictive features; based on performance metrics and model complexity trade-offs, the top 10 were selected for model development. These included reproductive history, age, tumor location, menopausal status, tumor diameter, lymphocyte count, platelet count, AFP, neutrophil count, and CEA.

### 2.6 Model construction

1. Support Vector Machine (SVM): This algorithm maximizes the interclass margin by identifying an optimal hyperplane to achieve data classification, making it well-suited for high-dimensional datasets.
2. Logistic Regression (LR): A generalized linear model that transforms linear regression outputs into probability values via a logistic (sigmoid) function, primarily applied to binary classification tasks.
3. Random Forest (RF): An ensemble learning method that constructs multiple decision trees and aggregates their predictions; it exhibits resistance to overfitting and is capable of handling high-dimensional data.
4. K-Nearest Neighbors (KNN): An instance-based learning algorithm that classifies samples by computing inter-sample distances and implementing a voting mechanism, suitable for multi-class classification tasks.
5. XGBoost: An ensemble learning algorithm based on gradient-boosted decision trees, characterized by robust predictive performance and strong generalization ability.

### 2.7 Machine learning interpretable tools

In this study, the model is interpreted using the SHAP method. SHAP is based on the Shapley values from game theory and is able to quantify the specific contribution of each feature to the model’s predicted outcomes and analyze the interactions between the features, providing an interpretable framework for model predictions. The unique SHAP value for each sample enables in-depth interpretation of individual prediction results.

### 2.8 Statistical analysis

All statistical modeling and visualizations were conducted using R software (version 4.4.2). Categorical variables were analyzed using the chi-square test or Fisher’s exact test and reported as frequencies (percentages). Continuous variables with a normal distribution were summarized as mean ± standard deviation, with intergroup comparisons conducted using the t-test. Non-normally distributed continuous variables were presented as quartiles, with groupwise variability assessed using the Wilcoxon rank-sum test. Statistical significance was defined as a two-tailed p-value < 0.05. The discriminatory performance of the model was quantified using the area under the receiver operating characteristic curve (AUC). For clinical utility assessment, decision curve analysis (DCA) was performed to calculate net benefit values across a range of risk thresholds, thereby evaluating the clinical decision-making utility of the predictive model.

## 3 Results

### 3.1 Patient characteristics

A total of 816 patients treated between January 2016 and December 2024 were included in the study, stratified into a training set (n=503), a test set (n=223), and a temporal validation set (n=90). The dataset exhibited a 6.3% missing value rate, which was addressed using Multiple Imputation. Baseline characteristics were well-balanced between the training and test sets, with no statistically significant differences observed (*p*>0.05). Detailed patient characteristics are presented in **Table 1**.

**Table 1:** Baseline characteristics of HR+ breast cancer patients in the training set and test set.

Group	All data	Train data	Test data	*P*-value
N	726	503	223	
Marital_Status				0.431
Single	6 (0.8%)	5 (1.0%)	1 (0.4%)	
Married	709 (97.7%)	492 (97.8%)	217 (97.3%)	
Divorced	4 (0.6%)	3 (0.6%)	1 (0.4%)	
Widowed	7 (1.0%)	3 (0.6%)	4 (1.8%)	
Parity				0.732
0	12 (1.7%)	10 (2.0%)	2 (0.9%)	
1	196 (27.0%)	141 (28.0%)	55 (24.7%)	
2	282 (38.8%)	191 (38.0%)	91 (40.8%)	
3	156 (21.5%)	106 (21.1%)	50 (22.4%)	
≥4	80 (11.0%)	55 (10.9%)	25 (11.2%)	
Menopause				0.259
No	385 (53.0%)	274 (54.5%)	111 (49.8%)	
Yes	341 (47.0%)	229 (45.5%)	112 (50.2%)	
BMI				0.775
<24	362 (49.9%)	247 (49.1%)	115 (51.6%)	
24-28	319 (43.9%)	223 (44.3%)	96 (43.0%)	
>28	45 (6.2%)	33 (6.6%)	12 (5.4%)	
Tumor_Location				0.806
Central	7 (1.0%)	5 (1.0%)	2 (0.9%)	
Upper outer quadrant	323 (44.5%)	216 (42.9%)	107 (48.0%)	
Upper inner quadrant	222 (30.6%)	159 (31.6%)	63 (28.3%)	
Lower inner quadrant	88 (12.1%)	62 (12.3%)	26 (11.7%)	
Lower outer quadrant	86 (11.8%)	61 (12.1%)	25 (11.2%)	
T_Stage				0.130
T1	238 (32.8%)	160 (31.8%)	78 (35.0%)	
T2	363 (50.0%)	247 (49.1%)	116 (52.0%)	
T3	125 (17.2%)	96 (19.1%)	29 (13.0%)	
Subtype				0.350
Luminal A	178 (24.5%)	118 (23.5%)	60 (26.9%)	
Luminal B	548 (75.5%)	385 (76.5%)	163 (73.1%)	
Grade				0.171
Well differentiated: I	132 (18.2%)	99 (19.7%)	33 (14.8%)	
Moderately differentiated: II	468 (64.5%)	323 (64.2%)	145 (65.0%)	
Poorly differentiated: III	126 (17.4%)	81 (16.1%)	45 (20.2%)	
Ki67				0.651
<14%	194 (26.7%)	131 (26.0%)	63 (28.3%)	
14%-30%	351 (48.3%)	242 (48.1%)	109 (48.9%)	
>30%	181 (24.9%)	130 (25.8%)	51 (22.9%)	
P53				0.619
No	451 (62.1%)	309 (61.4%)	142 (63.7%)	
Yes	275 (37.9%)	194 (38.6%)	81 (36.3%)	
HER2				0.168
No	600 (82.6%)	409 (81.3%)	191 (85.7%)	
Yes	126 (17.4%)	94 (18.7%)	32 (14.3%)	
ALNM				0.434
No	502 (69.1%)	343 (68.2%)	159 (71.3%)	
Yes	224 (30.9%)	160 (31.8%)	64 (28.7%)	
Age	46.89 ± 10.82	46.67 ± 10.74	47.38 ± 11.01	0.524
Neutrophil_Count	3.23 ± 1.30	3.26 ± 1.39	3.18 ± 1.06	0.715
Lymphocyte_Count	1.84 ± 0.57	1.82 ± 0.57	1.89 ± 0.59	0.168
Platelet_Count	207.97 ± 65.00	205.99 ± 64.22	212.42 ± 66.65	0.156
CEA	2.58 ± 2.72	2.52 ± 2.62	2.71 ± 2.92	0.283
AFP	2.54 ± 1.42	2.52 ± 1.41	2.60 ± 1.43	0.313

Among the included patients, the Luminal B subtype accounted for 75.5%, and 97.9% were married. Regarding reproductive history, A parity of 2 (P2) was the most common (38.8%), followed by P1 (27.0%) and P3 (21.5%). Overall, 47.0% of patients were postmenopausal, and 49.9% had a BMI <24 kg/m². Most tumors were located in the upper outer quadrant (44.5%), with T2 stage being the most prevalent (50.0%), and histological grading was predominantly moderately differentiated (Grade II, 64.5%). The Ki-67 index was most frequently 14–30% (48.3%), P53 positivity was observed in 37.9% of patients, and HER2 positivity in 17.4%. The incidence of axillary lymph node metastasis (ALNM) was 30.9%. The mean patient age was 46.9 ± 10.8 years. Median laboratory parameters were as follows: neutrophil count 3.23 × 10^9^/L, lymphocyte count 1.84 × 10^9^/L, platelet count 208.0 × 10^9^/L, carcinoembryonic antigen (CEA) 2.58 ng/mL, and alpha-fetoprotein (AFP) 2.54 ng/mL.

In the training set, between-group comparisons of ALNM status (**Table 2**) revealed that parity ≥3, postmenopausal status, tumor location in the upper outer quadrant, advanced tumor stage (T3), Luminal B subtype, poor histological grade (Grade III), high Ki67 index (>30%), older age, and elevated CEA levels were significantly associated with an increased risk of ALNM (*P* < 0.05). In contrast, variables including marital status and body mass index (BMI) showed no significant association with ALNM risk (*P*> 0.05).

**Table 2:** Clinical and pathological characteristics of the influence of the training set on the occurrence of ALNM.

Characteristic	NO ALNM	ALNM	*P*-value
N	343	160	
Marital_Status			0.387
Single	3 (0.9%)	2 (1.3%)	
Married	336 (98.0%)	156 (97.5%)	
Divorced	3 (0.9%)	0 (0.0%)	
Widowed	1 (0.3%)	2 (1.3%)	
Parity			<0.001
0	8 (2.3%)	2 (1.3%)	
1	116 (33.8%)	25 (15.6%)	
2	159 (46.4%)	32 (20.0%)	
3	48 (14.0%)	58 (36.3%)	
≥4	12 (3.5%)	43 (26.9%)	
Menopause			<0.001
No	234 (68.2%)	40 (25.0%)	
Yes	109 (31.8%)	120 (75.0%)	
BMI			0.886
<24	168 (49.0%)	79 (49.4%)	
24-28	151 (44.0%)	72 (45.0%)	
>28	24 (7.0%)	9 (5.6%)	
Tumor_Location			<0.001
Central	3 (0.9%)	2 (1.3%)	
Upper outer quadrant	122 (35.6%)	94 (58.8%)	
Upper inner quadrant	144 (42.0%)	15 (9.4%)	
Lower inner quadrant	37 (10.8%)	25 (15.6%)	
Lower outer quadrant	37 (10.8%)	24 (15.0%)	
T_Stage			<0.001
T1	131 (38.2%)	29 (18.1%)	
T2	168 (49.0%)	79 (49.4%)	
T3	44 (12.8%)	52 (32.5%)	
Subtype			<0.001
Luminal A	96 (28.0%)	22 (13.8%)	
Luminal B	247 (72.0%)	138 (86.3%)	
Grade			<0.001
Well differentiated: I	79 (23.0%)	20 (12.5%)	
Moderately differentiated: II	227 (66.2%)	96 (60.0%)	
Poorly differentiated: III	37 (10.8%)	44 (27.5%)	
Ki67			<0.001
<14%	109 (31.8%)	22 (13.8%)	
14%-30%	157 (45.8%)	85 (53.1%)	
>30%	77 (22.4%)	53 (33.1%)	
P53			>0.999
No	211 (61.5%)	98 (61.3%)	
Yes	132 (38.5%)	62 (38.8%)	
HER2			0.463
No	282 (82.2%)	127 (79.4%)	
Yes	61 (17.8%)	33 (20.6%)	
Age	44.06 ± 8.30	52.26 ± 13.02	<0.001
Neutrophil_Count	3.30 ± 1.40	3.17 ± 1.36	0.342
Lymphocyte_Count	1.83 ± 0.58	1.81 ± 0.54	0.974
Platelet_Count	210.41 ± 65.64	196.50 ± 60.17	0.028
CEA	2.23 ± 1.95	3.15 ± 3.59	0.011
AFP	2.47 ± 1.29	2.61 ± 1.64	0.777

### 3.2 Model construction and evaluation

**Figure 1:**
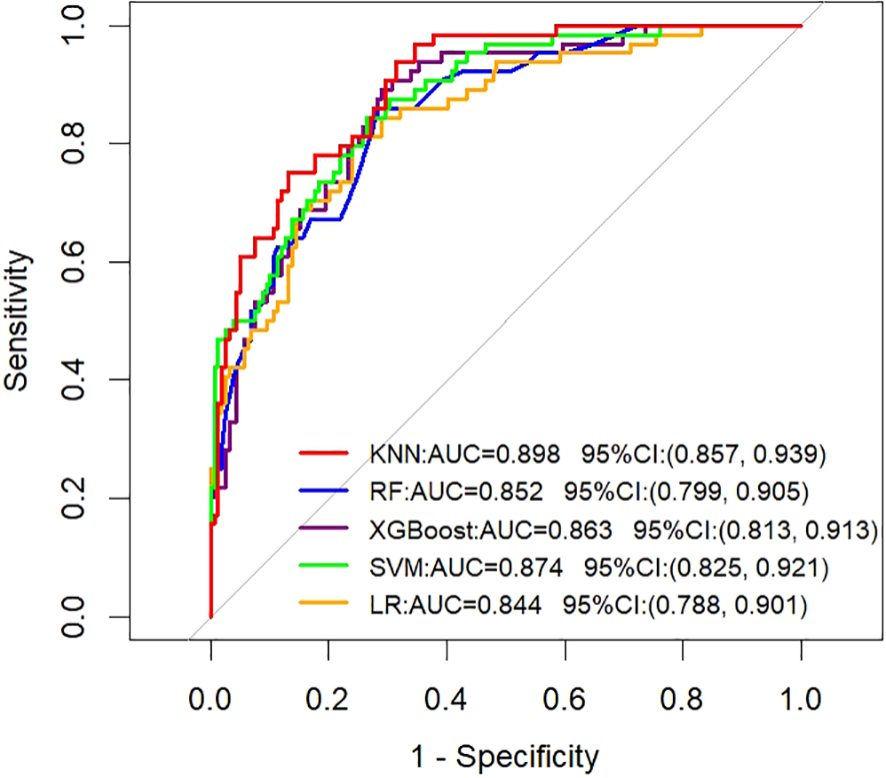
Test set: ROC curves analyzing the prediction performance of five ML algorithms for ALNM in HR+ patients.

Based on training set data, we developed five machine learning predictive models: XGBoost, RF, LR, SVM, and KNN. During model development, hyperparameter optimization was conducted using five-fold cross-validation combined with grid search to systematically evaluate the performance of each parameter combination. The test set and temporal validation set remained fully independent throughout the training and tuning process, and were only utilized once after model finalization to ensure an objective assessment of the model’s generalization ability. Evaluation of the test set demonstrated that all models achieved an AUC of >0.80 (**Figure 1**). Among these, the KNN model exhibited the strongest performance, with an AUC of 0.898 (95% confidence interval [CI]: 0.857–0.939).

**Figure 2:**
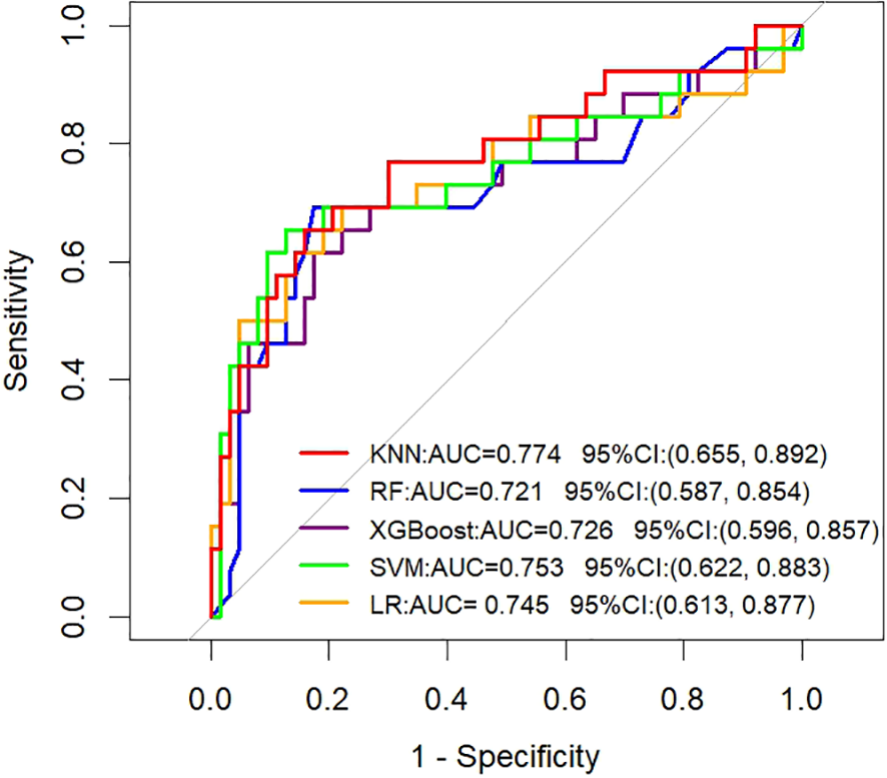
Temporal validation set: ROC curves analyzing the prediction performance of five ML algorithms for ALNM in HR+ patients.

**Figure 3:**
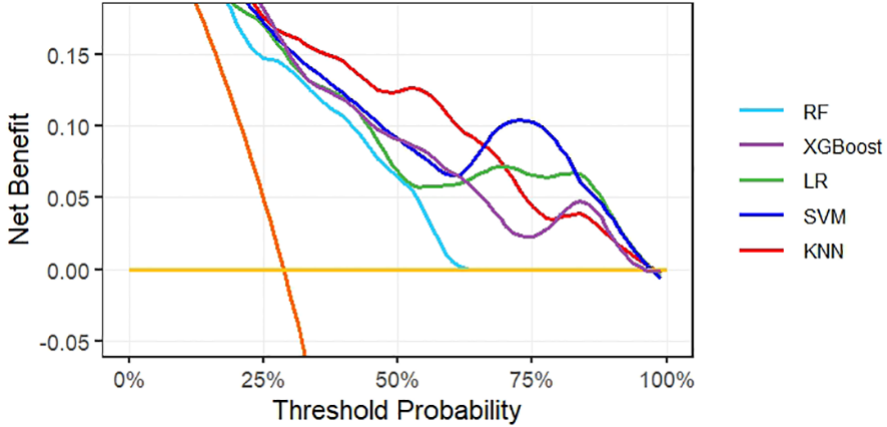
Decision curve analysis of net returns for five models with different threshold probabilities.

To evaluate the temporal generalizability of the models, we assessed their performance on the held-out temporal validation cohort (**Figure 2**). The KNN model exhibited robust performance on this temporal validation set, achieving an AUC of 0.774 (95% CI: 0.655–0.892). This outcome was highly consistent with its performance on the training set, confirming strong generalizability of the KNN model. To evaluate the clinical utility of the models, DCA was used to quantify the net clinical benefit of each model across a range of threshold probabilities (**Figure 3**). All models outperformed the reference strategies of “treating all patients” (orange line) and “treating no patients” (yellow line) on the decision curve. Notably, the KNN model yielded the highest net clinical benefit across a threshold probability range of 30–65%.

Model performance was further comprehensively assessed across multiple metrics, including accuracy, sensitivity, positive predictive value (PPV), negative predictive value (NPV), and F1 score (**Table 3**). These analyses confirmed the superior performance of the KNN model. Accordingly, the KNN model was designated as the optimal model in this study for predicting ALNM risk in patients with HR+ BC.

**Table 3:** Prediction performance of the model.

Model	AUC(%)	Sensitivity(%)	F1score	Accuracy(%)	PPV	NPV
KNN	0.898	0.641	0.694	0.838	0.760	0.864
RF	0.852	0.234	0.375	0.776	0.937	0.763
LR	0.844	0.547	0.583	0.775	0.625	0.826
SVM	0.874	0.578	0.627	0.803	0.685	0.840
XGBoost	0.863	0.546	0.614	0.802	0.700	0.832

### 3.3 Interpret the KNN model using the SHAP method

**Figure 4:**
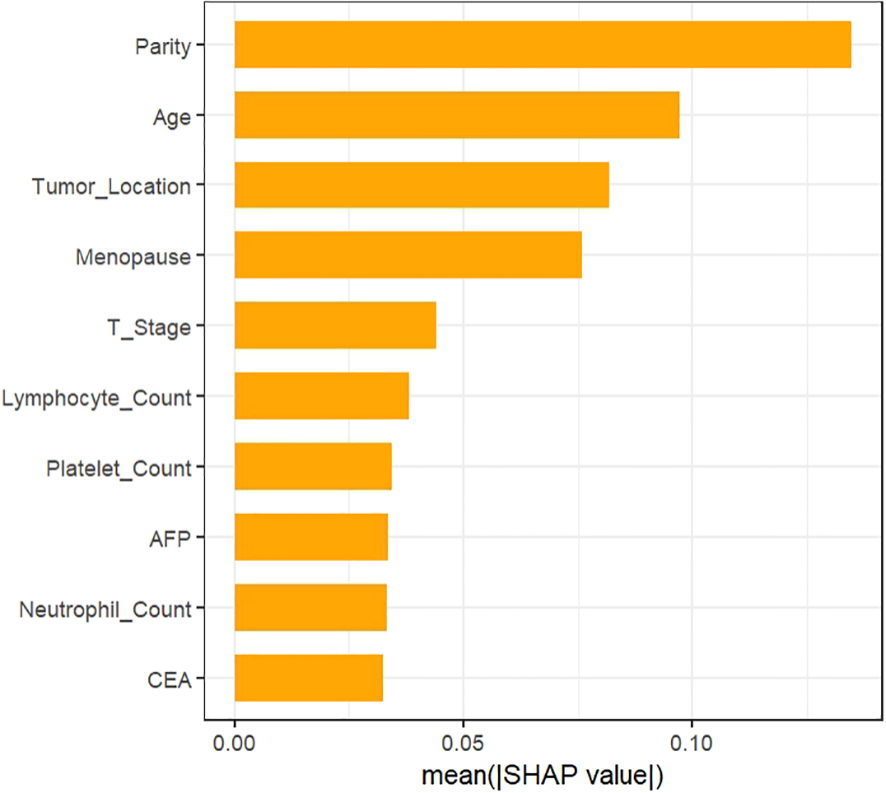
Weighting of variable importance.

**Figure 5:**
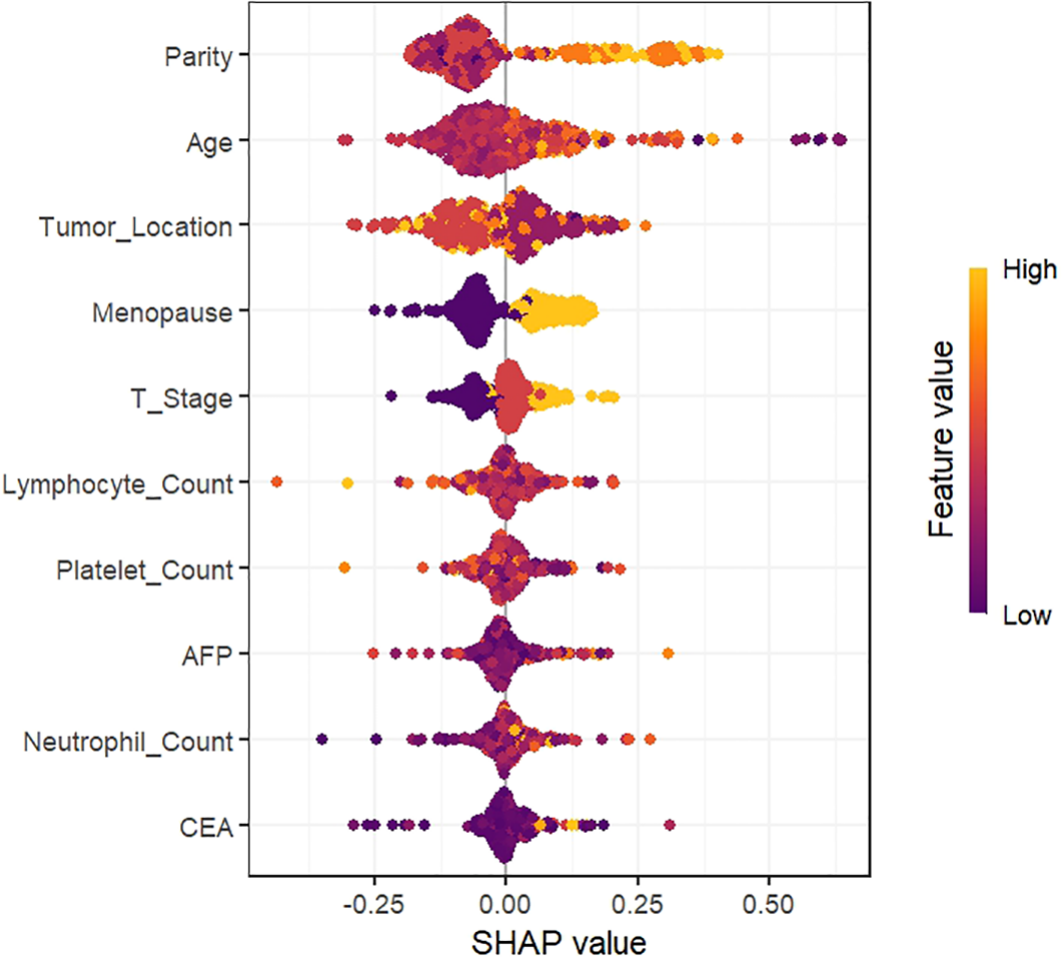
SHapley additive interpretation (SHAP) values.

Additionally, we ranked the relative importance of clinicopathologic features within the model. As depicted in **Figure 4**, reproductive history was identified as the most impactful predictor of ALNM development, followed by age, tumor location, menopausal status, tumor diameter, lymphocyte count, and platelet count. **Figure 5** further characterizes the directional effects of each variable on ALNM risk: positive SHAP values (orange, right) signify an elevated probability of ALNM, whereas negative values (purple, left) denote a reduced risk. Reproductive history exhibited a strong positive association with ALNM occurrence, where higher values (orange) correlated with elevated risk. Conversely, specific tumor locations (orange, left) were associated with favorable outcomes, as indicated by negative SHAP values. For instance, higher reproductive history scores (orange, right) correlated with poorer prognostic outcomes, whereas specific tumor locations (orange, left) were associated with more favorable outcomes relative to lower scores (purple, right).

### 3.4 SHAP waterfall diagram

**Figure 6:**
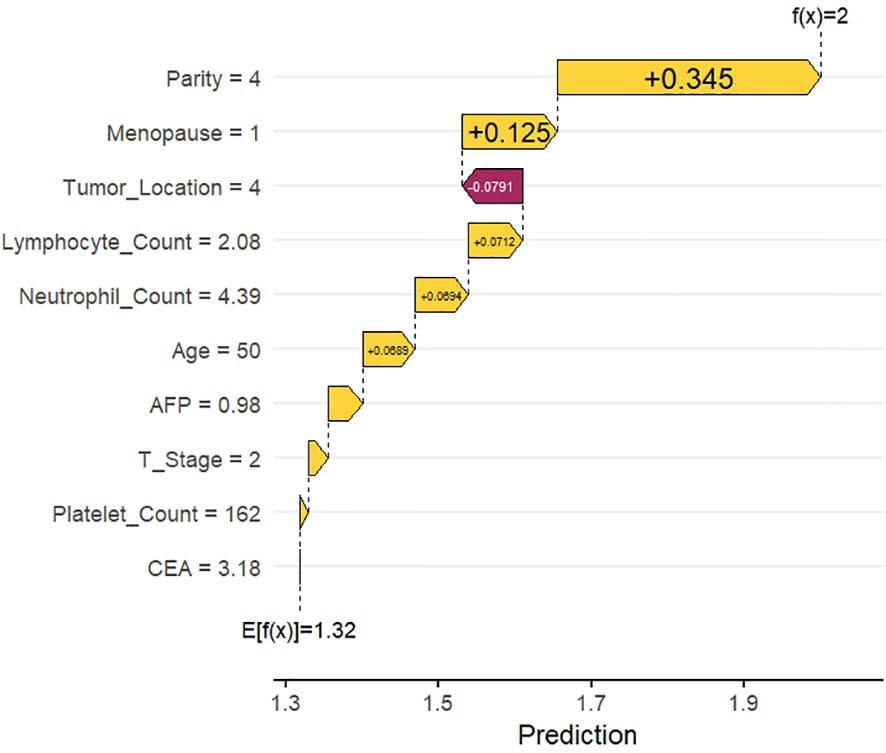
SHAP waterfall diagram for the occurrence of ALNM. Parity: none = 0, 1 = 1, 2 = 2, 3 = 3, >3 = 4; Menopause: not menopausal = 0, menopausal = 1; Tumor_Location: Upper outer quadrant = 1, Upper inner quadrant = 2, Lower inner quadrant = 3, Lower outer quadrant = 4, central = 0; T Stage: T1 = 1, T2 =2, T3 =3.

**Figure 7:**
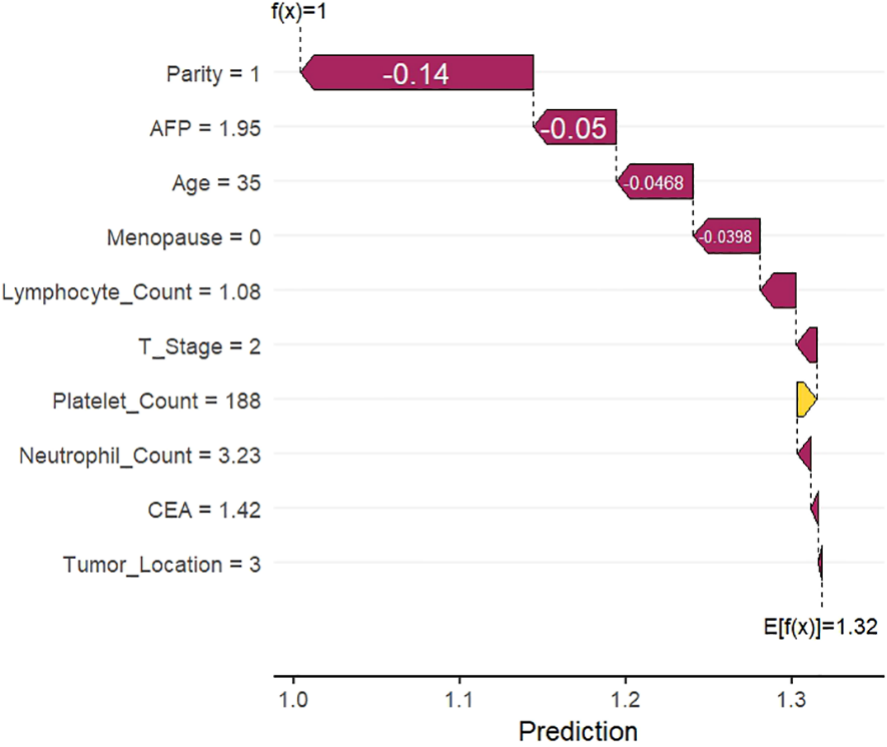
SHAP waterfall diagram without ALNM. Parity: none = 0, 1 = 1, 2 = 2, 3 = 3, >3 = 4; Menopause: not menopausal = 0, menopausal = 1; Tumor_Location: Upper outer quadrant = 1, Upper inner quadrant = 2, Lower inner quadrant = 3, Lower outer quadrant = 4, central = 0; T Stage: T1 = 1, T2 =2, T3 =3.

Individual SHAP waterfall plots for two randomly selected HR+ BC patients are presented in **Figure 6** (with ALNM) and **Figure 7** (without ALNM). Starting from the baseline predicted value (E[f(x)]), the plots sequentially illustrate the contribution of each feature to the final predicted value (f(x)). The bar corresponding to each feature quantifies its specific contribution to the prediction, where color indicates the direction of contribution (positive or negative) and length denotes the magnitude. In **Figure 6**, the predicted value increased from the baseline of 1.32 to 2 following the cumulative contributions of individual features. Specifically, reproductive history (P4) and menopausal status (postmenopausal) were the key positive contributors elevating the predicted value, significantly increasing the ALNM risk assessment. In contrast, the predicted value in **Figure 7** decreased from the baseline of 1.32 to 1, primarily driven by negative contributions from reproductive history (P1) and AFP levels (1.95 ng/mL), which reduced the ALNM risk assessment.

## 4 Discussion

For patients with cN0 BC, SLNB serves as the standard of care for evaluating axillary lymph node status. However, studies have reported that approximately 30% of cN0 patients harbor sentinel lymph node (SLN) metastases on SLNB (7). Notably, 20–60% of these patients with SLN metastases do not progress to further axillary lymph node involvement. For this subset, the clinical benefit of ALND remains uncertain, potentially constituting overtreatment (6). Thus, there is an unmet clinical need for reliable predictive tools to mitigate unnecessary axillary surgical interventions in cN0 patients—especially within the large HR+ subtype population.

In this study, the KNN model exhibited superior performance in predicting ALNM risk among cN0-stage HR+ BC patients compared to other machine learning algorithms. Leveraging the SHAP method, we quantified the relative importance of predictive features and elucidated the specific contribution of key variables to individual patient predictions via SHAP waterfall plots. By integrating the ranked feature importance with clinical expertise, we provide a data-driven foundation for individualized risk stratification and clinical decision-making in this patient population.

This research model confirmed reproductive history as the primary predictor of axillary lymph node metastasis. Multiple studies have pointed out that, after controlling for other factors, certain aspects of reproductive history remain independent predictors of lymph node metastasis. A long-term follow-up study based on the Swedish national population explicitly identified high parity (≥4 births) as an independent risk factor for axillary lymph node metastasis (OR = 1.56) (18). This confirms the universality of the association between reproductive history and lymph node metastasis from a large-scale population perspective. Multiple earlier studies provided corroborating evidence from different angles: Japanese research showed higher ALNM rates in BC patients with recent childbirth, particularly pronounced in HR+ patients (5); multivariate analysis of ALNM in invasive BC identified prior pregnancy history as a significant predictor of axillary metastasis (17); Another comparative study of Uyghur and Han Chinese women similarly confirmed multiple pregnancies as a shared risk factor (7). Zhang’s team’s large-scale Chinese cohort study confirmed a significant association between high parity and luminal B BC risk (19). The luminal B subtype itself is clinically recognized as closely associated with higher lymph node metastasis rates. A case-control study in Northeast China reached the same conclusion: multiple pregnancies increase BC risk (20).

We hypothesize that, within the cN0 HR+ population of this study, a history of high parity likely identifies a tumor subgroup with a biological predisposition toward luminal B characteristics and thus greater invasive potential. Recent cutting-edge research offers deeper mechanistic insights: a complete pregnancy-lactation cycle induces specific CD8^+^ T cells to persistently reside in breast tissue, thereby enduringly reshaping the immune microenvironment (21). Interestingly, this appears to contradict the epidemiological finding that “childbirth reduces BC incidence.” We propose a dual-role theory to reconcile this contradiction: at the population level, childbirth may reduce the risk of healthy cells becoming cancerous through tissue remodeling (preventive effect). However, once a tumor develops in breast tissue undergoing such remodeling, it may exhibit heightened invasiveness (promoting progression). This study focuses on the latter mechanism, revealing the significant predictive value of childbirth history during disease progression.

The current study identifies age, primary tumor location, and menopausal status as key predictors of ALNM in cN0 HR+ BC patients—findings that align with and extend prior clinical evidence. A comparative analysis of younger (<40 years) and older BC patients demonstrated that younger individuals had a higher rate of ALNM in non-metastatic disease (73.2% vs 55.6%, *P* < 0.001) and were more frequently diagnosed with T3/4 tumors (28.2% vs 13.8%, *P* < 0.001) (22). Additionally, multivariate analysis in a training cohort confirmed a significant association between younger age and increased ALNM risk (*P* = 0.006) (23), while Hu et al. further identified young age, together with tumor size, as independent predictors of ALNM (24). Regarding tumor location, Xiong et al. similarly reported age and upper outer quadrant tumor location as independent predictors of ALNM (11). In a study of T1-T2 N0 BC, the upper outer quadrant was specifically identified as a high-risk factor for ALNM (odds ratio [OR]=4.49, 95% CI: 1.63–12.37, *P* = 0.004) (25), and Xue et al. further confirmed tumor location (OR = 4.019, 95% CI: 1.304–12.383, *P* = 0.015) and tumor size (OR = 3.702, 95% CI: 1.517–9.034, *P* = 0.004) as independent risk factors (12). Furthermore, nomogram analyses consistently validated tumor location as an independent predictor of ALNM (*P* = 0.010) (26), with another nomogram-based study additionally recognizing preoperative tumor size (*P* = 0.030) and menopausal status (*P* = 0.017) as key prognostic factors (27). Consistent with our findings, prior studies have reported that postmenopausal women are at increased risk of axillary metastasis (28). We hypothesize that this may be attributed to the fact that breast masses in older women are often overlooked, leading to a longer disease course prior to diagnosis and thereby increasing the window of opportunity for ALNM development. Collectively, these data reinforce the clinical relevance of age, tumor location, and menopausal status in ALNM risk stratification, particularly in the cN0 HR+ population.

Tumor diameter is a core component of the TNM staging system, with tumors >5 cm in diameter typically associated with higher lymph node metastasis rates and increased distant metastasis risk (29). A study predicting ALNM using ultrasound features identified tumor diameter as a significant correlate (OR = 4.312, 95% CI: 2.933–7.364) (9). Gao et al. further demonstrated that tumor size (20–50 mm: OR = 3.682, 95% CI: 3.181–4.267, *p* < 0.001; >50 mm: OR = 9.725, 95% CI: 6.240–15.827, *p* < 0.001) and central tumor location (*p* < 0.001) were independent predictors of ALNM (8). Multiple other studies have consistently validated tumor size as a robust predictor of axillary lymph node status (30, 31). Consistent with these prior findings, our model confirms that tumor diameter, a key determinant of T stage, is a critical predictor of ALNM development in cN0 HR+ BC patients. This alignment underscores the biological rationale linking larger tumor burden to increased likelihood of lymphatic spread, reinforcing the clinical utility of tumor diameter in ALNM risk stratification.

Beyond clinicopathological characteristics, systemic inflammation, reflected by the preoperative neutrophil count, lymphocyte count, and platelet count, may offer specific prognostic insights in our cN0 HR+ cohort. Evidence suggests that neutrophil counts may serve as a predictor of ALNM in BC (32). This association may be underpinned by tumor-induced systemic reprogramming, where BC cells remotely alter bone marrow function via cytokines (e.g., IL-1β/G-CSF), promoting a pro-metastatic neutrophil phenotype (33). In HR+ patients, circulating neutrophils have been demonstrated to exhibit distinct molecular characteristics compared to other subtypes (34). Concurrently, a lower lymphocyte count may reflect weakened anti-tumor immune surveillance, further tipping the balance toward a pro-metastatic state. This is supported by a study in young women with HR+/HER2- breast cancer, which showed that higher stromal abundance of specific T−helper lymphocyte subsets is associated with improved survival outcomes (35). Similarly, platelets are activated by tumor-derived factors and can facilitate early metastasis by cloaking circulating tumor cells and secreting pro-angiogenic factors (36).

In summary, within the specific biological context of HR+ breast cancer, preoperative alterations in these three blood counts are not isolated phenomena. Instead, they may be interrelated components of a tumor-elicited systemic response. This accessible composite picture of host inflammation and immune status could provide a rationale for their collective predictive value in stratifying ALNM risk in cN0 patients.

Geng et al. identified the serum tumor marker CEA as an independent risk factor for ALNM (37), with patients with elevated CEA levels having a significantly higher risk of ALNM compared to those with normal levels (OR = 2.139, 95% CI: 1.261–3.630, *p* = 0.005) (38). It is important to note, however, that the predictive value of CEA for ALNM remains a subject of ongoing investigation with somewhat inconsistent conclusions across studies. While several studies, including those by Wu and Zhao, have supported its role as an independent predictor for nodal metastasis (38, 39), other analyses have found that CEA failed to retain independent significance in multivariate models (40). This discrepancy may stem from variations in study populations, sample sizes, or cutoff values. In the context of our cN0 HR+ cohort, the inclusion of CEA in the final model suggests a potential association, but its clinical application warrants careful interpretation and external validation.

Notably, the feature selection process in this study identified AFP as one of the predictors for ALNM. The significance of AFP may not stem from its role as a conventional diagnostic biomarker for BC, but rather from its potential as a surrogate signal indicating underlying aggressive biological behavior. This is supported by emerging research: an immunomics analysis specifically designed for BC observed differential expression of AFP, suggesting its non-incidental relevance in this context (41). Biologically, AFP is a cancer-embryonic protein with recognized immunomodulatory functions, such as suppressing NK cell and dendritic cell activity, thereby potentially fostering an immunosuppressive tumor microenvironment conducive to metastasis (42). Its re-expression in tumors may also reflect cellular dedifferentiation or stem cell-like properties, correlated with enhanced metastatic potential. Although not a standard marker, elevated serum AFP levels have been detected in a subset of BC patients and may correlate with postoperative recurrence risk (43, 44). Therefore, within our specific cohort of cN0 HR+ BC, preoperative AFP levels might provide unique supplementary information for risk stratification by hinting at both the immunosuppressive landscape and the inherent aggressiveness of tumor cells. Naturally, this association requires further validation in prospective, multicenter cohorts and basic experiments to clarify its causal mechanisms and feasibility for clinical application.

This study has several limitations. First, as a retrospective analysis from a single institution, the findings may be influenced by local clinical practices and patient demographics, affecting generalizability. Second, and most critically, our model was validated using a temporal validation cohort from the same hospital rather than a geographically independent external cohort. While this approach tests model stability over time and is stronger than a random split, it cannot assess geographic or demographic generalizability. Third, the sample size of the temporal validation cohort was relatively small, which may limit the precision of the performance estimates. Fourth, despite using multiple imputation, missing data in some variables could introduce bias. Finally, the clinical and biological implications of some predictor associations identified by the model, such as AFP, require further validation in independent cohorts and mechanistic investigation to fully establish their role in clinical decision-making.

## 5 Conclusion

In this study, we developed and validated a machine learning-based prediction model for ALNM in patients with cN0, HR+ BC. The KNN algorithm demonstrated the most robust performance among the models compared, with an AUC of 0.898 in the test set and 0.774 in the temporal validation cohort. Using the SHAP interpretability framework, parity was identified as the most influential preoperative predictor, followed by age, tumor location, and menopausal status. This interpretable model provides a data-driven approach for preoperative ALNM risk assessment in this specific patient population, offering insights that could inform discussions on axillary surgical management.

## Data Availability

The original contributions presented in the study are included in the article/supplementary material. Further inquiries can be directed to the corresponding author.
